# White Matter Microstructural Organization Is Higher with Age in Adult Superior Cerebellar Peduncles

**DOI:** 10.3389/fnagi.2016.00071

**Published:** 2016-04-14

**Authors:** Richard A. Kanaan, Matthew Allin, Marco M. Picchioni, Sukhwinder S. Shergill, Philip K. McGuire

**Affiliations:** ^1^Austin Health, Department of Psychiatry, University of MelbourneHeidelberg, VIC, Australia; ^2^Department of Psychosis Studies, Institute of Psychiatry, Psychology and Neuroscience, King’s College LondonLondon, UK; ^3^St Andrew’s Department, King’s College LondonNorthampton, UK

**Keywords:** fractional anisotropy, training, intelligence, aging, diffusion tensor imaging

## Abstract

Using diffusion tensor imaging, we conducted an exploratory investigation of the relationship between white matter tract microstructure and age in 200 healthy adult subjects using tract-based spatial statistics (TBSS). Though most tracts showed the slight decline in microstructural organization with age widely noted, in both superior cerebellar peduncles (SCP) it correlated positively with age, a result not previously reported. We confirmed this by using an alternative method, and by repeating our TBSS analysis in an additional sample of 133 healthy adults. In exploring this surprising result we considered the possibility that this might arise from the continual cognitive and motor refinement that is enacted in the cerebellum: we found that tract microstructure in both SCPs was also strongly correlated with IQ, again in contrast with all other tracts, and its relationship with age mediated by IQ, as a training model would predict.

## Introduction

The brain’s white matter undergoes marked change over the lifespan. It is progressively myelinated during childhood and adolescence (Chiang et al., 2011; Lebel and Beaulieu, 2011), dramatically improving its signal conduction; in old age there appears to be a more gradual deterioration in structure and corresponding function (Kochunov et al., 2009b; Glahn et al., 2013), perhaps due to decline in myelination (Kochunov et al., 2009a), though this is less well understood. The rates and timing of these changes vary across the brain, with associative tracts generally developing last and declining first (Kochunov et al., 2012).

One way of measuring these structural changes is with Diffusion Tensor Imaging (DTI), a neuroimaging modality that estimates the speed and direction of the diffusion of water in the brain using an MRI scanner (Basser et al., 1994). Scalar measures such as Fractional Anisotropy (FA) and Mean Diffusivity (MD) can be derived from the diffusion tensor, and microstructural features of the underlying brain white matter inferred from these (Basser and Pierpaoli, 1996).

A significant number of studies have now examined white matter aging using DTI – see (Bennett and Madden, 2014; Lockhart and DeCarli, 2014) for recent reviews. A moderately consistent picture of FA decline and MD increase with age has been observed in both cross sectional and longitudinal studies, and associations with the subtle decline in cognition in the healthy also widely reported (Bennett and Madden, 2014). The regional distribution of these findings is more complicated, with authors tending to focus on specific regions and little overlap between studies, but the decline appears to be strongest in frontal regions (Madden et al., 2012). In particular, though an anterior-posterior gradient in the decline of FA with age has been described (Madden et al., 2012; Bennett and Madden, 2014), there are remarkably few studies of posterior structures such as the cerebellar tracts (Burzynska et al., 2010).

We set out to explore the relationship between age and white matter microstructure across all white matter tracts in an initial sample of 200 healthy adults. We measured FA at its peak value along the tracts using tract-based spatial statistics (TBSS; Smith et al., 2006), and averaged these over 48 discrete tract regions. Given the surprising results of this initial analysis, we sought their confirmation using a different method [a region-of-interest (ROI) analysis], and their replication in an additional sample.

## Materials and Methods

### Subjects

Two hundred healthy subjects were selected from over 400 healthy volunteers recruited by the Institute of Psychiatry, London, as a demographically matched control group for a pathological sample. They had mean age (*SD*) 33.3 (12.6), range 17–63; 155 of them were male; and 190 of them were right handed. Mean (*SD*) IQ was 106.3 (13.8); years of education were 14.8 (2.8). They were 87% Caucasian, 5% African-Caribbean, 2% Asian, the remainder of mixed ethnicity. There were extensive missing values for the latter two measures (100 for ethnicity; 95 for years of education).

Given the results of the analysis of this first sample, a second sample was then obtained from the same register of volunteers, representing the remainder of the 400 who met the exclusion criteria below. This second sample had 133 subjects with mean age (*SD*) 35.4 (15.2), range 17–67; 25 were male; and 121 were right handed. Mean (*SD*) years of education were 15.6 (2.7); mean IQ was 112.4 (13.6). They were 92% Caucasian, 2% African-Caribbean, the remainder of mixed ethnicity. There were 37 missing values for ethnicity, 32 for years of education. For both samples, subjects were excluded if they had a history of any mental or neurological illness, head injury with loss of consciousness or drug/alcohol dependence. IQ was measured with the WAIS-III. All subjects gave written, informed consent after the study was explained to them, and the study was approved by the Institute of Psychiatry Research Ethics Committee.

### Image Acquisition and Pre-processing

Diffusion-weighted imaging data were acquired using a GE Signa 1.5 Tesla LX MRI system (General Electric, Milwaukee, WI, USA) with a standard birdcage quadrature head coil, using an echo planar imaging sequence peripherally gated to the cardiac cycle and optimized for the acquisition of white matter diffusion tensor MRI. Seven non-diffusion-weighted images (*b* = 0) were acquired, along with 64 images with diffusion gradients (*b* = 1300 s/mm2) uniformly distributed in space (Jones et al., 2002) at each of 60 slices. The TR was 15 cardiac R-R intervals with a TE of 107 ms. Whole-head acquisition gave isotropic (2.5 mm × 2.5 mm × 2.5 mm) voxels, reconstructed to a 1.875 × 1.875 mm in-plane pixel size. Following mutual-information image correction (diffusion images individually registered to the mean image see Catani et al., 2002), in-house software was used to remove non-brain tissue, determine the diffusion tensor using multivariate linear regression on log-transformed signal intensities, and calculate the FA in each remaining voxel (Basser et al., 1994).

### Tract-Based Spatial Statistics Analysis

This analysis was conducted using TBSS version 1.2 (Smith et al., 2006). FA images from all participants were aligned to the Johns Hopkins University – International Consortium of Brain Mapping DTI-81 white matter atlas (JHU DTI atlas) (Mori et al., 2008) using FMRIB’s non-linear image registration tool (FNIRT) in FSL^1^. The mean of the voxel-wise FA images was ‘skeletonised’ (to generate a study-specific mean FA ‘skeleton’ representing the centers of tracts common to all participants) and thresholded for white matter (at FA > 0.3). The aligned maps were then projected onto the mean white matter skeleton, and subdivided according to the 48 regions of the JHU DTI atlas, with FA averaged per region per-subject, and these regional means correlated with age using SPSS 22.0^2^. Alternative models (quadratic, cubic, exponential, logarithmic, exponential, logistic) of the relationship between FA and age were also explored using SPSS, using R-squared as a measure of model-fit.

### Region-of-Interest Analysis

FA and MD scans were first normalized using a two-stage process: a study-specific template was first created, and then the FA and MD images registered to it. To create the study-specific template, the mean *b* = 0 image from every subject was registered using SPM2 (Wellcome Department of Imaging Neuroscience, London, UK) to the SPM2 EPI template. The derived mapping parameters for each subject were then applied to that subject’s FA image. These normalized FA images were themselves averaged, and smoothed with an 8 mm Gaussian kernel. The FA images were then registered to this new template, again using SPM2, and the registration parameters applied to the MD. The registered FA images were segmented [using the default tissue probability information (‘priors’) in SPM2] and these probabilistic maps thresholded at 10% probability to generate liberal white matter masks. The registered FA and MD images were then smoothed with a 5 mm kernel, before masking to create white-matter-only FA and MD maps. A 2 mm spherical ROI was placed by sight on the Left Superior Cerebellar Peduncle (SCP) on the white matter template (**Figure 2**), and values (peak and mean) extracted from the FA and MD maps using XBAM_v4 (Institute of Psychiatry, London).

## Results

Repeated-measures ANOVA of the TBSS tract-averages from our initial sample found a main effect of age (*F* = 2.25, df = 47; *p* < 0.001, Greenhouse–Geisser corrected for non-sphericity), an effect of tract (*F* = 878.6, df = 15.13; *p* < 0.001), and a significant tract-by-age interaction (*F* = 1.4, df = 711.5; *p* < 0.001). *Post hoc* tract-age Pearson correlations were examined, revealing a strong correlation of bilateral SCP FA with age, which was both the strongest of all tracts bar the fornix and, remarkably, was positive, bilaterally (0.39 left, 0.364 right, both at *p* < 0.001, Benjamini–Hochberg corrected for multiple comparisons; Hochberg and Benjamini, 1990), when every other significant tract correlation was negative (Supplementary Table S1).

**FIGURE 1:**
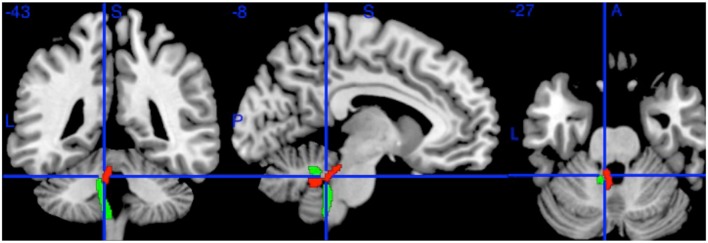
**The Left Superior Cerebellar Peduncle (red) and Left Inferior Cerebellar Peduncle (green) from the Johns Hopkins University DTI atlas.** The blue crosshairs indicate the center of the 2 mm spherical region of interest (at MNI coordinates -8, -43, -27). Additional ROIs (not shown) were drawn on the Right Superior Cerebellar Peduncle at (6, -43, -27) and Left Inferior Cerebellar Peduncle (-10, -42, -36).

Can this be true, when a substantial body of research (Madden et al., 2012; Bennett and Madden, 2014) shows that white matter integrity generally declines with age? A number of alternative explanations for this surprising result suggest themselves. Firstly, it could be that this result occurred because of some artifact arising from our method. We therefore repeated the analysis in the same sample but with a different method, sampling the SCPs using a conventional ROI drawn on the inner part of the SCPs on images co-registered using a different approach (**Figure 1**). Taking the mean FA of each ROI, the correlation was still positive but somewhat weaker (left, *r* = 0.19; *p* = 0.007; right, *r* = 0.17; *p* = 0.014), which is not unexpected given the smaller ROI (Kanaan et al., 2006). An ROI drawn on an adjacent control region (left inferior cerebellar peduncle) showed no age correlation (*r* = 0, *p* = 0.96).

Secondly, it could be that this was simply a biased sample, or a chance result. To explore this we looked at an additional sample of 133 healthy controls: the same relationship was seen to hold, with bilateral SCP FA having the strongest relationship, positively correlated with age (controls: 0.375, left and 0.339, right, both at *p* < 0.001, Benjamini–Hochberg corrected), in contrast with all other tracts, which either had no significant correlation with age or showed a decline (Supplementary Table S2).

**FIGURE 2:**
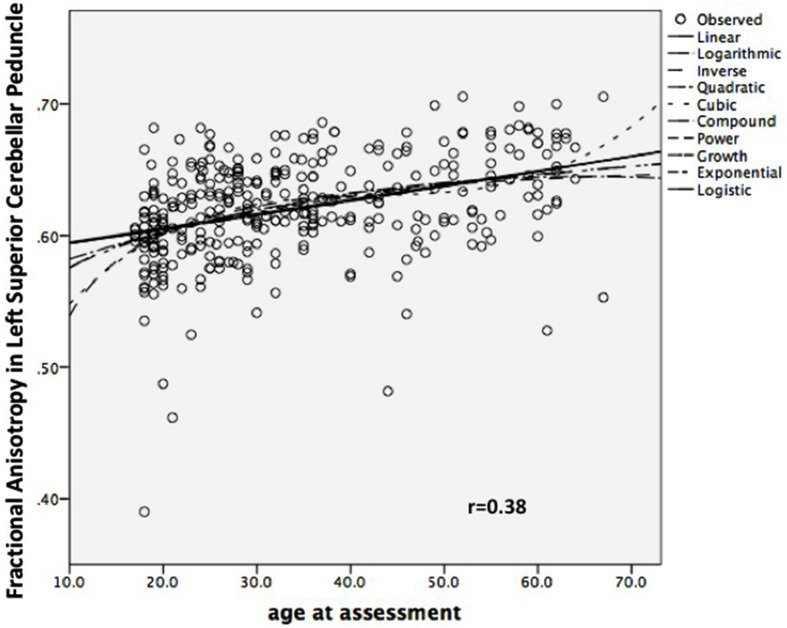
**Scatterplot of Left Superior Cerebellar Peduncle Fractional Anisotropy against Age, combined sample (*n* = 333)**.

It could be that a linear model is a poor approximation for what is really a more complex relationship – but a positive linear relationship does not appear inappropriate on visual inspection, and no other shape yielded a better fit, in particular (**Figure 2**).

It could be that this result occurred in both samples because of some artifact arising from our method. We therefore repeated the analysis, with a different method, sampling the SCPs using a conventional ROI drawn on the inner part of the SCPs on images co-registered using a different approach (**Figure 2**). Taking the mean FA of each ROI, the correlation was still positive but somewhat weaker (left, *r* = 0.19; *p* = 0.007; right, *r* = 0.17; *p* = 0.014), which is not unexpected given the smaller ROI (Kanaan et al., 2006).

Finally, it could be that this was a partial volume effect (the inclusion of voxels encompassing cerebro-spinal fluid as well as white matter) in this peri-ventricular tract. We have four reasons for thinking this an unlikely explanation of our findings. Firstly, TBSS suffers from relatively little partial volume artifact due to its selection of cross-sectional maxima (Metzler-Baddeley et al., 2012). Secondly, the confirmatory ROI above was placed on the inner part of the tract, distant from the ventricles, precisely to minimize any partial volume effect, yet returned an equivalent result. Thirdly, other peri-ventricular tracts, such as the adjacent inferior cerebellar peduncles (see **Figure 1**), did not show positive correlations with age. And fourthly, as the ventricles, including the 4th ventricle, enlarge with age (Salami et al., 2012), this would tend to increase partial volume, and so decrease measured FA with age – the opposite of what we found.

To further explore the nature of these white matter differences we repeated the ROI analysis on the MD maps, *post hoc*, finding no correlation, positive or negative, of MD with age in the SCP (left: *r* = 0.09, *p* = 0.2).

We investigated *post hoc* the relationship of age and FA with IQ, the one cognitive measure we had in a substantial number (202) of the combined sample. We found highly significant correlations of both SCPs with full-scale IQ (*r* = 0.286, left, *p* < 0.001; *r* = 0.242, right, *p* = 0.001), again in contrast with every other tract (Benjamini–Hochberg corrected; Supplementary Table S3), confirming the critical role of the cerebellum in cognition (Broussard, 2013). More importantly, such cognitive indices are now recognized to mediate the effects of training on brain changes with age, perhaps through such capacities as cognitive reserve (Salthouse, 2006; Lustig et al., 2009; Yaffe et al., 2009). A mediating role for IQ would therefore offer some support for the idea that training was at least partly responsible for the FA-age relationship (Madden et al., 2012), and a partial mediation of the age-FA relationship by IQ was what we observed: Age correlated to IQ (0.289, *p* < 0.001); IQ correlated to Left SCP (0.286, *p* < 0.001); Age correlated to Left SCP (0.356, *p* < 0.001); Age still correlated to Left SCP, once IQ was controlled, but at a reduced strength (0.298, *p* < 0.001) suggesting partial mediation (Baron and Kenny, 1986). The verbal and performance subscales separately showed the same pattern.

## Discussion

We found strong positive correlations between age and FA in the bilateral SCPs, in contrast with all other tracts. We confirmed this surprising result in an additional sample, and in the original sample using an additional method. We found, further, that this relationship had no corresponding relationship with MD, but was partially mediated by IQ.

If our finding was true, how could it be undiscovered, and what could it mean? One answer to the former may lie in the tendency by researchers to focus on the cerebrum: we could find only two large studies (Burzynska et al., 2010; Coutu et al., 2014) reporting any relationship of cerebellar white matter microstructure and aging in adults. Neither found an increase in SCP with age, though both were smaller than our study, and with differing sample ages (Burzynska and colleagues had a group of young, and a group of elderly subjects, Coutu and colleagues a range from 33 to 85). Nevertheless, a relative sparing of ‘posterior’ and ‘inferior’ structures in FA decline with age has been widely noted (Madden et al., 2012; Bennett and Madden, 2014). In terms of its meaning, though we accept that our results are both surprising and unexplained, we note that motor and cognitive training have been shown to selectively increase FA in healthy adults (Bengtsson et al., 2005; Scholz et al., 2009; Lovden et al., 2010), and the cerebellum is one of the main seats of training (Broussard, 2013). Though this sample was not selected on the basis of any specific training, they will have had the naturalistic exposure of honing their motor and cognitive skills that is common to all, and will continue to some degree across the lifespan. This does take some support from our finding of mediation by IQ, but can only convincingly be demonstrated by longitudinal studies.

There are many determinants of FA in healthy subjects, including axon diameter and packing density, but the two main determinants are myelination and tract coherence – the extent to which the tract fibers run in a coordinated manner, rather than crossing each other (Beaulieu, 2002). As an increase in myelination would be associated with a decrease in MD, which we did not find, this suggests that what differs over the adult lifespan is not the degree of myelination but the extent of the SCP’s organization. Though short term training is commonly, though not invariably, associated with a decrease in MD, suggestive of an increase in myelination (Burzynska et al., 2010) we note ours is a cerebellar rather than cerebral tract, and reflects, if our hypothesis is correct, not the result of targeted, intensive training, but the gradual refinement of the tract’s connections over a lifetime.

This hypothesis is limited by our study’s cross-sectional nature – any speculation about intra-individual changes cannot find confirmation from this data. The study is also limited by the biases in sampling that are inherent to this approach: though subjects were healthy, they were volunteers in mental-health-related projects, and will not represent the population as a whole. Their ‘healthiness’ is also not absolute, of course: though they had no mental or neurological illness and had grossly normal MRI scans, there was no screening for systemic illnesses such as diabetes or cerebro-vascular disease, which remain unexplored potential confounds of the relationship between age and white matter microstructure.

## Conclusion

The field of diffusion imaging has been fairly consistent in describing subtle declines in indices of white matter microstructure across the adult lifespan, but they have largely ignored the cerebellum. We have found a striking contrast to this decline in the main cerebellar output tract, with white matter organization found to be higher with age. This resisted our attempts to falsify it, and we are encouraged in our explanation of this as the effect of lifelong cognitive and motor training by the associations we found with intelligence. If correct, this may represent an encoding of the refinement of human cognitive and motor experience in white matter.

## Author Contributions

All authors listed have made substantial, direct and intellectual contribution to the work, and approved it for publication.

## Conflict of Interest Statement

The authors declare that the research was conducted in the absence of any commercial or financial relationships that could be construed as a potential conflict of interest.

## Footnotes

1 http://fsl.fmrib.ox.ac.uk/fsl/fslwiki/

2 www.ibm.com/software/analytics/spss
