# Supplementary material for: White Matter Microstructural Organization Is Higher with Age in Adult Superior Cerebellar Peduncles
Source: Front Aging Neurosci. 2016 Apr 14;8:71. doi: 10.3389/fnagi.2016.00071 (PMC4830843; doi:10.3389/fnagi.2016.00071)
Supplement: Supplementary file 1 [file Data_Sheet_1.DOCX]

**Supplementary Table 1: Correlations of Fractional Anisotropy with Age in 48 brain white matter tracts, first sample (n=200).**

| **Tract Name** | **Correlation with age** | **p-value** |
| --- | --- | --- |
| Anterior Corona Radiata L | -0.319^*^ | <0.001 |
| Anterior Corona Radiata R | -0.333^*^ | <0.001 |
| Anterior Limb Internal Capsule L | 0.039 | 0.586 |
| Anterior Limb Internal Capsule R | 0.020 | 0.779 |
| Body Corpus Callosum | -0.161 | 0.023 |
| Cerebral Peduncle L | -0.281^*^ | <0.001 |
| Cerebral Peduncle R | -0.286^*^ | <0.001 |
| Cingulum/Hippocampus L | -0.022 | 0.757 |
| Cingulum/Hippocampus R | 0.019 | 0.790 |
| Cingulum L | -0.121 | 0.089 |
| Cingulum R | -0.055 | 0.440 |
| Corticospinal tract L | -0.051 | 0.473 |
| Corticospinal tract R | -0.090 | 0.207 |
| External Capsule L | -0.098 | 0.169 |
| External Capsule R | -0.144 | 0.041 |
| Fornix | -0.451^*^ | <0.001 |
| Fornix/stria terminalis L | -0.192^*^ | 0.006 |
| Fornix/stria terminalis R | -0.224^*^ | 0.001 |
| Genu Corpus Callosum | -0.151 | 0.032 |
| Inferior Cerebellar Peduncles L | -0.144 | 0.042 |
| Inferior Cerebellar Peduncles R | -0.122 | 0.086 |
| Medial Lemniscus L | 0.089 | 0.210 |
| Medial Lemniscus R | 0.118 | 0.095 |
| Middle Cerebellar Peduncles | -0.034 | 0.631 |
| Pontine crossing tracts | -0.010 | 0.883 |
| Posterior Corona Radiata L | -0.306^*^ | <0.001 |
| Posterior Corona Radiata R | -0.259^*^ | <0.001 |
| Posterior Limb Internal Capsule L | -0.214^*^ | 0.002 |
| Posterior Limb Internal Capsule R | -0.236^*^ | 0.001 |
| Posterior Thalamic Radiations L | -0.269^*^ | <0.001 |
| Posterior Thalamic Radiations R | -0.236^*^ | 0.001 |
| Retrolenticular Internal Capsule L | -0.236^*^ | 0.001 |
| Retrolenticular Internal Capsule R | -0.247^*^ | <0.001 |
| Sagittal Stratum/ILF L | -0.025 | 0.727 |
| Sagittal Stratum/ILF R | -0.078 | 0.275 |
| Splenium Corpus Callosum | -0.051 | 0.470 |
| Superior Cerebellar Peduncles L | 0.390^*^ | <0.001 |
| Superior Cerebellar Peduncles R | 0.364^*^ | <0.001 |
| Superior Corona Radiata L | -0.268^*^ | <0.001 |
| Superior Corona Radiata R | -0.334^*^ | <0.001 |
| Superior Fronto-occipital Fasciculus L | 0.027 | 0.704 |
| Superior Fronto-occipital Fasciculus R | -0.004 | 0.955 |
| Superior Longitudinal Fasciculus L | -0.174^*^ | 0.014 |
| Superior Longitudinal Fasciculus R | -0.151 | 0.033 |
| Tapetum L | 0.023 | 0.749 |
| Tapetum R | -0.028 | 0.693 |
| Uncinate L | 0.014 | 0.840 |
| Uncinate R | 0.120 | 0.091 |

L=Left, R=Right; ILF=Inferior Longitudinal Fasciculus

^*^Significant after Benjamini-Hochberg Correction for Multiple Comparisons

**Supplementary Table 2: Correlations of Fractional Anisotropy with Age in 48 brain white matter tracts, second sample (n=133).**

| **Tract Name** | **Correlation with age** | **p-value** |
| --- | --- | --- |
| Anterior Corona Radiata L | -0.320^*^ | <0.001 |
| Anterior Corona Radiata R | -0.316^*^ | <0.001 |
| Anterior Limb Internal Capsule L | 0.067 | 0.444 |
| Anterior Limb Internal Capsule R | 0.064 | 0.461 |
| Body Corpus Callosum | 0.002 | 0.982 |
| Cerebral Peduncle L | -0.171 | 0.050 |
| Cerebral Peduncle R | -0.279^*^ | 0.001 |
| Cingulum/Hippocampus L | 0.065 | 0.459 |
| Cingulum/Hippocampus R | 0.022 | 0.803 |
| Cingulum L | 0.057 | 0.517 |
| Cingulum R | 0.007 | 0.939 |
| Corticospinal tract L | 0.087 | 0.320 |
| Corticospinal tract R | 0.015 | 0.861 |
| External Capsule L | -0.007 | 0.937 |
| External Capsule R | 0.014 | 0.872 |
| Fornix | -0.345^*^ | <0.001 |
| Fornix/stria terminalis L | -0.081 | 0.351 |
| Fornix/stria terminalis R | -0.110 | 0.208 |
| Genu Corpus Callosum | -0.161 | 0.064 |
| Inferior Cerebellar Peduncles L | -0.061 | 0.485 |
| Inferior Cerebellar Peduncles R | -0.020 | 0.817 |
| Medial Lemniscus L | 0.137 | 0.116 |
| Medial Lemniscus R | 0.200 | 0.021 |
| Middle Cerebellar Peduncles | 0.132 | 0.129 |
| Pontine crossing tracts | -0.046 | 0.598 |
| Posterior Corona Radiata L | -0.121 | 0.166 |
| Posterior Corona Radiata R | -0.090 | 0.302 |
| Posterior Limb Internal Capsule L | -0.036 | 0.680 |
| Posterior Limb Internal Capsule R | -0.282^*^ | 0.001 |
| Posterior Thalamic Radiations L | -0.284^*^ | 0.001 |
| Posterior Thalamic Radiations R | -0.257^*^ | 0.003 |
| Retrolenticular Internal Capsule L | -0.186 | 0.032 |
| Retrolenticular Internal Capsule R | -0.249^*^ | 0.004 |
| Sagittal Stratum/ILF L | -0.027 | 0.760 |
| Sagittal Stratum/ILF R | -0.134 | 0.125 |
| Splenium Corpus Callosum | 0.026 | 0.762 |
| Superior Cerebellar Peduncles L | 0.375^*^ | <0.001 |
| Superior Cerebellar Peduncles R | 0.339^*^ | <0.001 |
| Superior Corona Radiata L | -0.195 | 0.024 |
| Superior Corona Radiata R | -0.240^*^ | 0.005 |
| Superior Fronto-occipital Fasciculus L | -0.013 | 0.882 |
| Superior Fronto-occipital Fasciculus R | -0.011 | 0.897 |
| Superior Longitudinal Fasciculus L | -0.045 | 0.610 |
| Superior Longitudinal Fasciculus R | -0.069 | 0.428 |
| Tapetum L | 0.042 | 0.627 |
| Tapetum R | -0.074 | 0.398 |
| Uncinate L | 0.081 | 0.356 |
| Uncinate R | 0.121 | 0.165 |

L=Left, R=Right; ILF=Inferior Longitudinal Fasciculus

^*^Significant after Benjamini-Hochberg Correction for Multiple Comparisons

**Supplementary Table 3: Correlations of Fractional Anisotropy with IQ in 48 brain white matter tracts, combined sample (n=202).**

| **Tract Name** | **Correlation with IQ** | **p-value** |
| --- | --- | --- |
| Anterior Corona Radiata L | 0.043 | 0.545 |
| Anterior Corona Radiata R | 0.040 | 0.573 |
| Anterior Limb Internal Capsule L | 0.067 | 0.341 |
| Anterior Limb Internal Capsule R | 0.094 | 0.181 |
| Body Corpus Callosum | 0.029 | 0.678 |
| Cerebral Peduncle L | -0.012 | 0.868 |
| Cerebral Peduncle R | 0.018 | 0.796 |
| Cingulum/Hippocampus L | 0.089 | 0.208 |
| Cingulum/Hippocampus R | 0.130 | 0.066 |
| Cingulum L | -0.038 | 0.596 |
| Cingulum R | -0.026 | 0.713 |
| Corticospinal tract L | 0.071 | 0.316 |
| Corticospinal tract R | 0.042 | 0.550 |
| External Capsule L | 0.068 | 0.338 |
| External Capsule R | 0.088 | 0.213 |
| Fornix | -0.130 | 0.065 |
| Fornix/stria terminalis L | 0.027 | 0.707 |
| Fornix/stria terminalis R | 0.067 | 0.340 |
| Genu Corpus Callosum | -0.002 | 0.975 |
| Inferior Cerebellar Peduncles L | 0.116 | 0.101 |
| Inferior Cerebellar Peduncles R | 0.121 | 0.085 |
| Medial Lemniscus L | 0.138 | 0.050 |
| Medial Lemniscus R | 0.158 | 0.025 |
| Middle Cerebellar Peduncles | 0.026 | 0.710 |
| Pontine crossing tracts | 0.080 | 0.255 |
| Posterior Corona Radiata L | 0.031 | 0.659 |
| Posterior Corona Radiata R | 0.063 | 0.370 |
| Posterior Limb Internal Capsule L | -0.017 | 0.813 |
| Posterior Limb Internal Capsule R | -0.092 | 0.191 |
| Posterior Thalamic Radiations L | 0.013 | 0.859 |
| Posterior Thalamic Radiations R | 0.033 | 0.640 |
| Retrolenticular Internal Capsule L | -0.045 | 0.528 |
| Retrolenticular Internal Capsule R | -0.037 | 0.600 |
| Sagittal Stratum/ILF L | 0.072 | 0.309 |
| Sagittal Stratum/ILF R | 0.057 | 0.424 |
| Splenium Corpus Callosum | -0.001 | 0.990 |
| Superior Cerebellar Peduncles L | 0.286^*^ | <0.001 |
| Superior Cerebellar Peduncles R | 0.242^*^ | 0.001 |
| Superior Corona Radiata L | -0.026 | 0.715 |
| Superior Corona Radiata R | 0.015 | 0.828 |
| Superior Fronto-occipital Fasciculus L | 0.088 | 0.215 |
| Superior Fronto-occipital Fasciculus R | 0.120 | 0.088 |
| Superior Longitudinal Fasciculus L | 0.150 | 0.033 |
| Superior Longitudinal Fasciculus R | 0.094 | 0.185 |
| Tapetum L | <0.001 | 0.995 |
| Tapetum R | -0.012 | 0.863 |
| Uncinate L | -0.052 | 0.461 |
| Uncinate R | 0.046 | 0.515 |

L=Left, R=Right; ILF=Inferior Longitudinal Fasciculus

^*^Significant after Benjamini-Hochberg Correction for Multiple Comparisons
